# A pathway linking pulse pressure to dementia in adults with Down syndrome

**DOI:** 10.1093/braincomms/fcae157

**Published:** 2024-05-09

**Authors:** Batool Rizvi, Patrick J Lao, Mithra Sathishkumar, Lisa Taylor, Nazek Queder, Liv McMillan, Natalie C Edwards, David B Keator, Eric Doran, Christy Hom, Dana Nguyen, H Diana Rosas, Florence Lai, Nicole Schupf, Jose Gutierrez, Wayne Silverman, Ira T Lott, Mark Mapstone, Donna M Wilcock, Elizabeth Head, Michael A Yassa, Adam M Brickman

**Affiliations:** Center for the Neurobiology of Learning and Memory, University of California, Irvine, Irvine, CA 92697, USA; Department of Neurobiology and Behavior, University of California, Irvine, Irvine, CA 92697, USA; Taub Institute for Research on Alzheimer’s Disease and the Aging Brain, College of Physicians and Surgeons, Columbia University, New York, NY 10032, USA; Department of Neurology, College of Physicians and Surgeons, Columbia University, New York, NY 10032, USA; Gertrude H. Sergievsky Center, College of Physicians and Surgeons, Columbia University, New York, NY 10032, USA; Center for the Neurobiology of Learning and Memory, University of California, Irvine, Irvine, CA 92697, USA; Department of Neurobiology and Behavior, University of California, Irvine, Irvine, CA 92697, USA; Center for the Neurobiology of Learning and Memory, University of California, Irvine, Irvine, CA 92697, USA; Department of Neurobiology and Behavior, University of California, Irvine, Irvine, CA 92697, USA; Center for the Neurobiology of Learning and Memory, University of California, Irvine, Irvine, CA 92697, USA; Department of Neurobiology and Behavior, University of California, Irvine, Irvine, CA 92697, USA; Center for the Neurobiology of Learning and Memory, University of California, Irvine, Irvine, CA 92697, USA; Department of Neurobiology and Behavior, University of California, Irvine, Irvine, CA 92697, USA; Taub Institute for Research on Alzheimer’s Disease and the Aging Brain, College of Physicians and Surgeons, Columbia University, New York, NY 10032, USA; Department of Neurology, College of Physicians and Surgeons, Columbia University, New York, NY 10032, USA; Gertrude H. Sergievsky Center, College of Physicians and Surgeons, Columbia University, New York, NY 10032, USA; Department of Psychiatry and Human Behavior, University of California, Irvine, Irvine, CA 92697, USA; Department of Pediatrics, University of California, Irvine, Orange, CA 92688, USA; Department of Pediatrics, University of California, Irvine, Orange, CA 92688, USA; Department of Pediatrics, University of California, Irvine, Orange, CA 92688, USA; Department of Neurology, Massachusetts General Hospital, Harvard University, Boston, MA 02114, USA; Department of Radiology, Athinoula Martinos Center, Massachusetts General Hospital, Harvard University, Charlestown, MA 02129, USA; Department of Neurology, Massachusetts General Hospital, Harvard University, Boston, MA 02114, USA; Taub Institute for Research on Alzheimer’s Disease and the Aging Brain, College of Physicians and Surgeons, Columbia University, New York, NY 10032, USA; Department of Neurology, College of Physicians and Surgeons, Columbia University, New York, NY 10032, USA; Gertrude H. Sergievsky Center, College of Physicians and Surgeons, Columbia University, New York, NY 10032, USA; Department of Epidemiology, Mailman School of Public Health, Columbia University, New York, NY 10032, USA; Department of Neurology, College of Physicians and Surgeons, Columbia University, New York, NY 10032, USA; Department of Pediatrics, University of California, Irvine, Orange, CA 92688, USA; Department of Pediatrics, University of California, Irvine, Orange, CA 92688, USA; Department of Neurology, University of California, Irvine, Irvine, CA 92697, USA; Department of Neurology, Indiana University School of Medicine, Indianapolis, IN 46202, USA; Department of Pathology and Laboratory Medicine, University of California, Irvine, Irvine, CA 92697, USA; Center for the Neurobiology of Learning and Memory, University of California, Irvine, Irvine, CA 92697, USA; Department of Neurobiology and Behavior, University of California, Irvine, Irvine, CA 92697, USA; Taub Institute for Research on Alzheimer’s Disease and the Aging Brain, College of Physicians and Surgeons, Columbia University, New York, NY 10032, USA; Department of Neurology, College of Physicians and Surgeons, Columbia University, New York, NY 10032, USA; Gertrude H. Sergievsky Center, College of Physicians and Surgeons, Columbia University, New York, NY 10032, USA

**Keywords:** pulse pressure, Down syndrome, white matter hyperintensities, aging, Alzheimer’s disease

## Abstract

Adults with Down syndrome are less likely to have hypertension than neurotypical adults. However, whether blood pressure measures are associated with brain health and clinical outcomes in this population has not been studied in detail. Here, we assessed whether pulse pressure is associated with markers of cerebrovascular disease and is linked to a diagnosis of dementia in adults with Down syndrome via structural imaging markers of cerebrovascular disease and atrophy. The study included participants with Down syndrome from the Alzheimer’s Disease - Down Syndrome study (*n* = 195, age = 50.6 ± 7.2 years, 44% women, 18% diagnosed with dementia). Higher pulse pressure was associated with greater global, parietal and occipital white matter hyperintensity volume but not with enlarged perivascular spaces, microbleeds or infarcts. Using a structural equation model, we found that pulse pressure was associated with greater white matter hyperintensity volume, which in turn was related to increased neurodegeneration, and subsequent dementia diagnosis. Pulse pressure is an important determinant of brain health and clinical outcomes in individuals with Down syndrome despite the low likelihood of frank hypertension.

## Teaser

Rizvi *et al*. found that increased pulse pressure is associated with small vessel cerebrovascular disease in adults with Down syndrome. They also found that pulse pressure was indirectly related to dementia through intermediate markers of white matter hyperintensities and neurodegeneration.

## Graphical Abstract

**Graphical Abstract:**
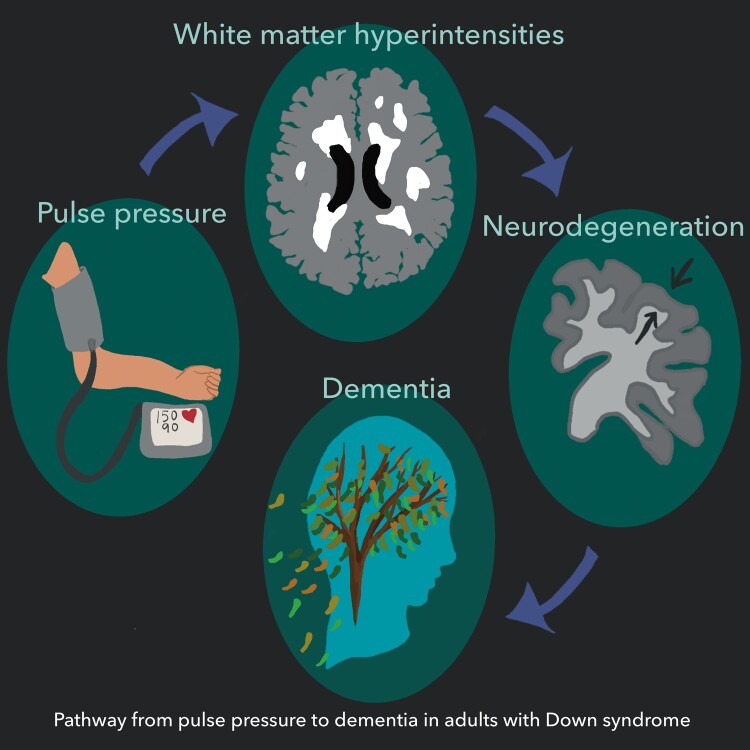

## Introduction

As individuals with Down syndrome (DS) reach 50 years of age, nearly all have sufficient beta-amyloid and tau pathology to meet the pathological criteria for Alzheimer’s disease.^1-5^ Despite the inevitable accumulation of beta-amyloid and tau pathology in adults with DS, there is some variability in the age of onset of clinical symptoms of dementia and in the severity and the course of decline of clinical symptoms.^2,4,6^ The factors that account for the variability in clinical onset and course are poorly understood. Although Alzheimer’s disease pathogenesis in the context of DS is typically attributed to a single pathway model (i.e. the amyloid cascade hypothesis)^7-9^ and the clinical course is typically framed according to the amyloid-tau-neurodegeneration (ATN) biomarker classification scheme,^10,11^ there is increasing evidence of the contribution of cerebrovascular disease to both clinical and pathogenic progression.^12-15^ Despite relatively low prevalence of classical vascular risk factors,^16,17^ individuals with DS have cerebrovascular abnormalities on MRI, including white matter hyperintensities (WMH), enlarged perivascular spaces (PVS), microbleeds and infarcts, which increase across Alzheimer’s disease-related clinical diagnoses.^18^

In the neurotypical population, the presence and severity of vascular risk factors, particularly high blood pressure or hypertension, lead to cerebrovascular disease, which in turn is associated with neurodegeneration, cognitive decline and risk and progression of clinical Alzheimer’s disease.^19-21^ These relationships have not been examined systematically among adults with DS, likely due to the low prevalence of vascular risk factors.^17^ Individuals with DS have lower blood pressure than individuals from the neurotypical population,^22-24^ as well as lower prevalence and incidence of hypertension.^25^ The physiological mechanisms of these observations are not well understood. Nonetheless, it is possible that increases in blood pressure measurements may be associated with poorer brain outcomes among older adults with DS even if those measurements are within the normal range for the neurotypical population. In support of this hypothesis, studies in the neurotypical population found that elevated blood pressure even in non-hypertensive adults is associated with worse cognition.^26,27^

In the current study, we examined the association of common clinical blood pressure-related measurements of vascular health with MRI markers of small vessel disease or dysfunction and downstream diagnosis of dementia in older adults with DS. Systolic blood pressure (SBP) measures the force of the heart exerting on artery walls at each beat, while diastolic blood pressure (DBP) measures the force of the heart exerting on the artery walls in between beats, reflecting peripheral resistance. Pulse pressure (PP), or the difference between systolic and diastolic blood pressure, is an indicator of arterial stiffness. Mean arterial pressure (MAP) is the average arterial pressure during one cardiac cycle.^28^ In the neurotypical population, hypertension is related to increased risk for stroke, WMH, infarcts and Alzheimer’s disease-related pathology.^29^ Increased PP can lead to atherosclerosis even in normotensive individuals,^30^ which is associated with WMH.^31,32^ Higher PP is also associated with medial temporal lobe atrophy in Alzheimer’s disease.^33^ Compared with other measures of blood pressure, PP is more tightly linked to Alzheimer’s disease pathology.^34,35^ Higher PP can affect cerebral blood flow and grey matter and white matter integrity, increasing the risk for dementia.^36,37^

Given the documented stronger associations of clinical outcomes with PP than other blood pressure indicators,^34,38^ we focused primarily on this measure in the current analyses. We assessed the associations of blood pressure measures with MRI markers of cerebrovascular disease. Previously, we showed that regional WMH burden is associated with medial temporal lobe atrophy in community-dwelling older adults.^39-41^ Prior work also demonstrated that greater posterior WMH burden is associated with a diagnosis of dementia in adults with DS and in autosomal dominant Alzheimer’s disease.^18,42^ Based on these observations, we hypothesized that increased PP is related to greater WMH, which in turn is related to lower cortical thickness, and subsequently to dementia.

## Materials and methods

### Participants

This study included participants from the Alzheimer’s Disease - Down Syndrome (ADDS; U01 AG051412) study, which characterizes the factors that contribute to the development of Alzheimer’s disease dementia among older adults with DS. Participants were enrolled at multiple sites, including Columbia University/New York State Institute for Basic Research in Developmental Disabilities (*n* = 54), Massachusetts General Hospital/Harvard Medical School (*n* = 75) and University of California, Irvine (*n* = 66). Participants whose blood pressure was measured during their baseline visit were included in the analyses (*N* = 195).

The study was approved by the institutional review boards of the participating institutions, and written informed consent was obtained from the participants and/or their legal guardian or legally authorized representative. We received assent from every participant before every procedure.

### Blood pressure measures

Blood pressure, including systolic and diastolic blood pressure, was assessed within 3 months of the MRI visit. Blood pressure was assessed within a single measurement while the participant was seated. The blood pressure measurement device was not specified; sites used a standardized automatic blood pressure cuff used in their centre or a sphygmomanometer when the automatic cuff was not available. Pulse pressure was derived by taking the difference between SBP and DBP: PP = SBP − DBP. MAP was calculated using the following equation: MAP = (SBP + 2(DBP))/3. We used a structured interview, the Health History Review of Systems, to determine whether participants had a clinical history of hypertension or hypotension. If these diagnoses were endorsed, we asked whether the participant was receiving treatment, which was coded dichotomously (yes/no).

### Clinical diagnosis

As part of the diagnostic procedure, participants underwent neuropsychological testing to assess cognition in domains typically affected by Alzheimer’s disease. Study personnel reviewed clinical charts, conducted interviews with knowledgeable informants and conducted a standardized clinical and neurological examination. A consensus panel of clinicians, expert in the diagnosis of dementia in individuals with DS, assigned a final diagnosis based on the information collected, which did not include any biomarker data.^43^ One of four Alzheimer’s disease-related diagnoses was assigned to each participant: cognitively stable (CS), mild cognitive impairment-DS (MCI-DS), possible Alzheimer’s disease dementia, and definite Alzheimer’s disease dementia. For the current analyses, we categorized individuals into two groups: with Alzheimer’s disease dementia (including possible and definite Alzheimer’s disease dementia) or without dementia (including CS or MCI-DS). A diagnosis of CS indicated the absence of clinically significant cognitive decline, MCI-DS indicated subtle cognitive decline over time beyond what was expected with age but not severe enough to indicate dementia. Possible Alzheimer’s disease dementia indicated some symptoms of dementia present but with inconsistent evidence of progression. Definite Alzheimer’s disease dementia indicated substantial evidence of cognitive and associated functional decline with high degree of confidence.

### MRI

**Figure 1:**
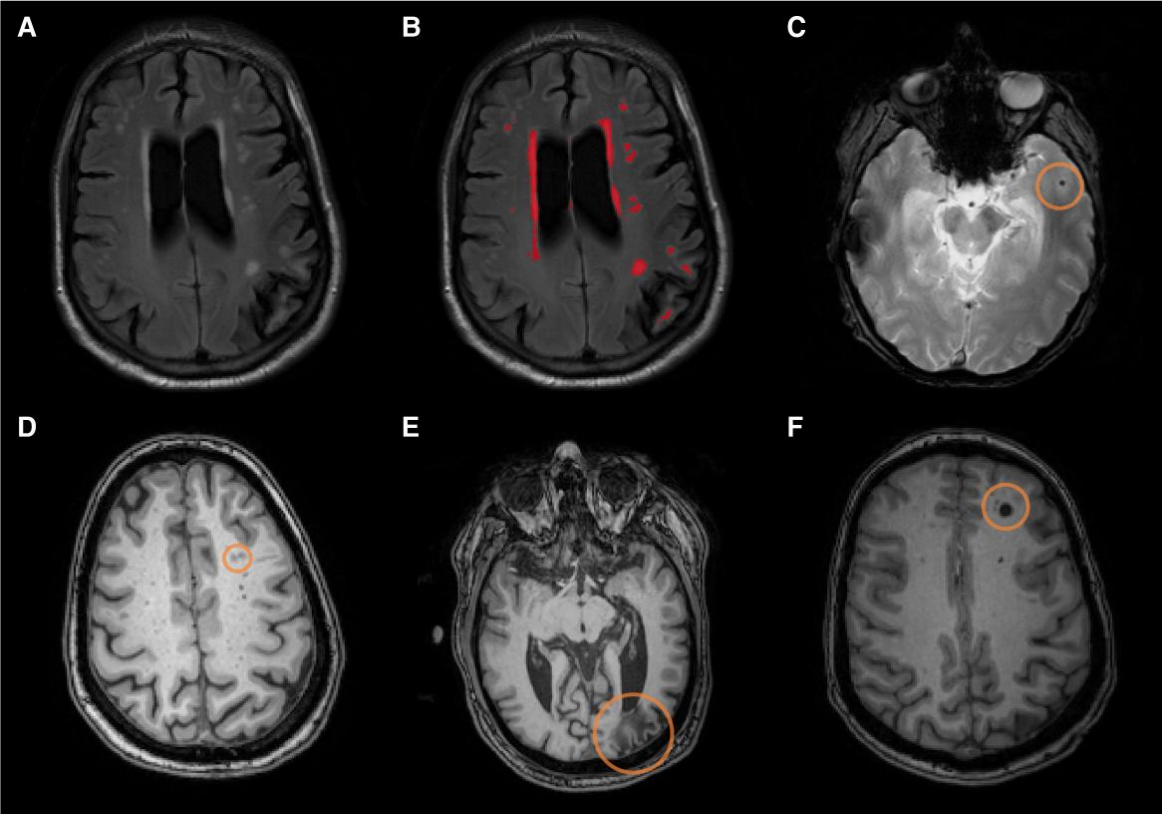
**MRI markers of cerebrovascular disease included in the study.** (**A**) Unlabelled WMH on an axial FLAIR image. (**B**) Labelled WMH in red on an axial FLAIR image. (**C**) Microbleed circled on an axial T_2_*-GRE. (**D**) Perivascular spaces circled on an axial T_1_-weighed image. (**E**) Distal cortical infarct circled on an axial T_1_-weighted image. (**F**) Deep infarct circled on an axial T_1_-weighted image.

MRI scans (*n* = 145) were acquired on a Siemens Prisma 3T at Columbia University (*n* = 30) and MGH (*n* = 58) or a Philips Achieva 3T at UC Irvine (*n* = 57). The Alzheimer’s Disease Neuroimaging Initiative MRI protocol was used at all sites [T_1_-weighted scan: repetition time (TR)/echo time (TE)/inversion time (TI): 2300/2.96/900 ms; voxel size: 1 × 1 × 1 mm^3^; T_2_-weighted fluid attenuated inversion recovery (FLAIR) scan (TR/TE/TI: 5000/386/1800 ms; voxel size: 0.4 × 0.4 × 0.9 mm^3^); and a T_2_-*-weighted gradient echo (GRE) scan (TR/TE: 650/20 ms; voxel size: 0.8 × 0.8 × 4 mm^3^) or susceptibility weighted image (SWI: TRE/TE: 27/30 ms; voxel size: 0.9 × 0.9 × 1.5 mm^3^)]. Total and regional (by cerebral lobe) WMH volumes were quantified from T_2_-weighted FLAIR scans, using a previously described method (see Fig. 1A and B).^18,44^ Microbleeds were counted by visual inspection, as hypointense round or ovoid lesions in deep and/or lobar regions on GRE or SWI, and were globally scored as present or not (see Fig. 1C). Enlarged PVS were visually rated as hypointensities on T_1_ scans across 13 brain regions and rated from 0 to 2 (see Fig. 1D). These ratings were then summed for a global score ranging from 0 (no enlarged PVS in any region) to 26 (severe enlarged PVS in all regions).^45^ Infarcts were visually counted on FLAIR scans as discrete hypointense lesions (>5 mm) with partial or complete hyperintense ring and confirmed on T_1_ scans (hypointense areas) and were globally scored as present or not (see Fig. 1E and F).

### Volume and cortical thickness

Each participant’s T_1_-weighted image was processed with FreeSurfer v.6.0 (http://surfer.nmr.mgh.harvard.edu/*)*. We calculated hippocampal volume by averaging left and right hippocampal volume, dividing by estimated total intracranial volume and multiplying the value by 1000. We calculated the Alzheimer’s disease cortical signature by averaging left and right entorhinal cortical thickness, parahippocampal cortex, inferior parietal lobe, pars opercularis, pars orbitalis, pars triangularis, inferior temporal lobe, temporal pole, precuneus, supramarginal gyrus, superior parietal lobe and superior frontal lobe.

### Statistical analysis

Separate general linear models were used to test the association of PP with regional WMH and enlarged PVS score. We used separate logistic regression models to test the association between PP and the presence of any microbleed and any infarct. All regression analyses were adjusted for age, sex/gender, and scanner type. In a sensitivity analysis, we tested whether associations remained after removing participants who were treated for either hypertension or hypotension. Associations with SBP, DBP and MAP are reported in supplementary results.

Structural equation model (SEM) analyses were conducted in R (version 4.3.0) through RStudio, using ‘lavaan’. We tested whether PP is associated with greater WMH and whether WMH is related to increased neurodegeneration and subsequently to dementia. We also tested whether PP is directly related to dementia. The latent variable of WMH was estimated by the four regional distributions of WMH (i.e. frontal WMH, temporal WMH, parietal WMH, occipital WMH), and the latent variable of neurodegeneration was estimated by hippocampal volume and Alzheimer’s disease cortical signature thickness. Pulse pressure and dementia were included as single indicator variables. Prior to performing the SEM analysis, all neuroimaging variables were log transformed with an added constant of 0.01. We used full information maximum likelihood estimation and allowed the loadings of the first manifest variables of each factor to be estimated freely. We assessed the overall model fit to be ‘acceptable’ if *X*^2^  *P* > 0.05, Comparative fit index (CFI ≥ 0.90), Tucker–Lewis index (TLI ≥ 0.90), root mean square error of approximation (RMSEA < 0.08) and standardized root mean square residual (SRMR < 0.08).^46^ Because the chi-square test is sensitive to sample size, we considered other measures of model fit more heavily. We conducted this SEM analysis without age as a covariate and report results when including age in the supplementary results.

## Results

One-hundred ninety-five participants were included. Table 1 displays summary characteristics, including demographic, clinical and imaging variables, and the sample size associated with each variable. About 17% of the participants with DS were characterized as having a high blood pressure reading (SBP ≥ 130 and/or DBP ≥ 80 mmHg), while around 28% demonstrated a low blood pressure reading (SBP < 90 and/or DBP < 60 mmHg).

**Table 1:** Sample demographic characteristics and summary variables

Characteristic	Values
*N*	195
Age, mean (SD) years	50.58 (7.19)
Sex, *n* (%) women	85 (43.6)
Diagnostic category (*n* = 187)	
Cognitively stable, *n* (%)	110 (56.4)
MCI-DS, *n* (%)	42 (21.5)
Dementia, *n* (%)	35 (17.9)
Blood pressure measure, mmHg (*n* = 195)	
Pulse pressure, mean (SD)	44.01 (12.57)
Systolic blood pressure, mean (SD)	110.16 (14.57)
Diastolic blood pressure, mean (SD)	66.15 (10.36)
Mean arterial pressure, mean (SD)	80.81 (10.36)
High blood pressure reading^a^, *n* (%)	33 (16.9)
Low blood pressure reading^a^, *n* (%)	55 (28.2)
High pulse pressure reading^b^, *n* (%)	19 (9)
Treated for hypertension, *n* (%)	2 (1)
Treated for hypotension, *n* (%)	9 (4.6)
WMH volume, cm^3^ (*n* = 123)	
Total WMH, mean (SD)	3.93 (1.93)
Frontal WMH, mean (SD)	1.43 (1.98)
Temporal WMH, mean (SD)	0.51 (0.71)
Parietal WMH, mean (SD)	0.54 (0.16)
Occipital WMH, mean (SD)	0.94 (1.31)
Enlarged PVS score, mean (*n* = 110)	9.19 (5.57)
Presence of microbleeds, *n* (%) (*n* = 92)	13 (6.7)
Presence of infarcts, *n* (%) (*n* = 113)	20 (10.3)
Regional volume/thickness (*n* = 125)	
Average hippocampal volume (TIV adj.), mean (SD)	2.35 (0.38)
Average Alzheimer’s disease cortical signature thickness, mean (SD)	2.75 (0.16)

^a^Based on baseline SBP and DBP and the general guidelines for hypertension (SBP ≥ 130 and/or DBP ≥ 80 mmHg) and hypotension (SBP < 90 and/or DBP < 60 mmHg).

^b^Included when pulse pressure reading was >60 mmHg.

### Association of pulse pressure with cerebrovascular imaging markers

**Figure 2:**
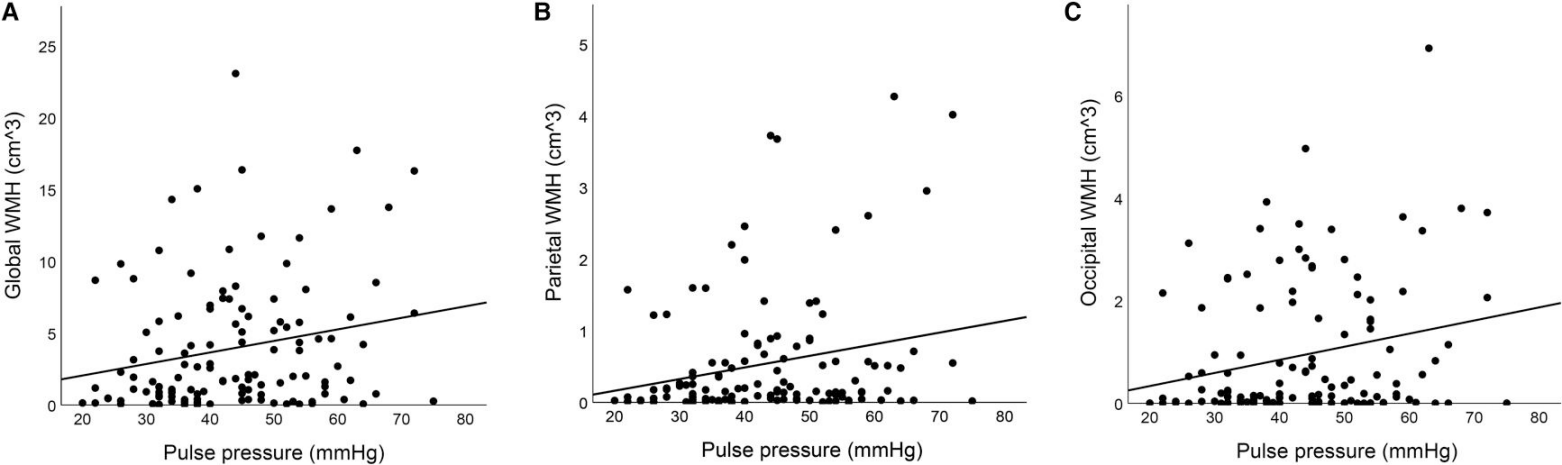
**Scatterplots of associations between PP with WMH volumes.** Scatterplots displaying bivariate associations between (**A**) PP and global WMH, (**B**) PP and parietal WMH and (**C**) PP and occipital WMH. Results of these associations with covariate adjustment are displayed in Table 2.

Higher PP was associated with greater global WMH, parietal WMH and occipital WMH (see Table 2 and Fig. 2). Of the covariates, age was associated with higher frontal, parietal and occipital WMH. Sex/gender was not associated with any of the WMH measures. Scanner type was associated with all of the WMH measures. In a sensitivity analysis, after removing participants who were treated for either hypotension or hypertension, higher PP remained associated with greater global, parietal and occipital WMH volumes (see Supplemental Table 1). Systolic blood pressure, DBP and MAP were not associated with global or regional WMH volumes (see Supplemental Table 2). Pulse pressure was not associated with PVS, microbleeds or infarcts (see Table 2). Similarly, SBP, DBP and MAP were also not associated with PVS, microbleeds or infarcts (see Supplemental Table 2).

**Table 2:** Associations of PP and covariates with cerebrovascular imaging markers

Predictor	Outcome measure	*B*	95% CI	β	t/Wald^a^	*P*-value
Pulse pressure	Global WMH	0.057	(0.001, 0.114)	0.154	2.002	0.048
	Frontal WMH	0.012	(−0.015, 0.039)	0.073	0.871	0.385
	Temporal WMH	0.008	(−0.001, 0.018)	0.140	1.697	0.092
	Parietal WMH	0.012	(0.001, 0.024)	0.172	2.139	0.035
	Occipital WMH	0.018	(0.003, 0.034)	0.172	0.023	0.023
	Microbleeds^b^	−0.012	(0.942, 1.037)	0.998	0.234	0.629
	Perivascular spaces	0.073	(−0.008, 0.155)	0.164	1.781	0.078
	Infarcts^b^	0.019	(0.979, 1.062)	1.019	0.874	0.350
Age	Global WMH	0.124	(0.012, 0.227)	0.186	2.376	0.019
	Frontal WMH	0.066	(0.017, 0.115)	0.227	2.672	0.009
	Temporal WMH	0.004	(−0.013, 0.022)	0.040	0.485	0.629
	Parietal WMH	0.022	(0.002, 0.043)	0.174	2.132	0.035
	Occipital WMH	0.043	(0.014, 0.072)	0.224	2.953	0.004
	Microbleeds^b^	0.089	(0.994, 1.203)	1.093	3.354	0.067
	Enlarged perivascular spaces	0.265	(0.110, 0.420)	0.325	3.396	<0.001
	Infarcts^b^	0.083	(1.003, 1.176)	1.086	4.148	0.042
Sex/gender	Global WMH	−0.542	(−1.970, 0.887)	−0.058	−0.751	0.454
	Frontal WMH	−0.038	(−0.716, 0.641)	−0.009	−0.110	0.912
	Temporal WMH	−0.056	(−0.297, 0.184)	−0.038	−0.464	0.644
	Parietal WMH	−0.019	(−0.306, 0.267)	−0.011	−0.135	0.893
	Occipital WMH	−0.343	(−0.745, 0.059)	−0.127	−1.690	0.094
	Microbleeds^b^	0.272	(0.364, 4.735)	1.313	0.173	0.678
	Enlarged perivascular spaces	0.082	(−1.982, 2.146)	0.007	0.079	0.937
	Infarcts^b^	0.688	(0.652, 6.075)	1.990	1.461	0.227
Scanner type	Global WMH	4.286	(2.900, 5.671)	0.469	6.126	<0.001
	Frontal WMH	1.370	(0.712, 2.028)	0.343	4.124	<0.001
	Temporal WMH	0.620	(0.387, 0.853)	0.430	5.265	<0.001
	Parietal WMH	0.717	(0.440, 0.995)	0.409	5.119	<0.001
	Occipital WMH	1.213	(0.823, 1.603)	0.458	6.160	<0.001
	Microbleeds^b^	0.436	(0.448, 5.337)	1.546	0.476	0.490
	Enlarged perivascular spaces	1.140	(−0.887, 3.168)	0.099	1.115	0.267
	Infarcts^b^	0.517	(0.979, 1.062)	1.677	0.957	0.227

Separate primary regression models: outcome measure ∼ pulse pressure + age + sex/gender + scanner type + intercept.

^a^A *t*-value is shown for linear regressions, and a Wald statistic is shown for logistic regressions.

^b^Logistic regression was applied for dichotomous outcomes. β = exponential (β) and 95% CI = 95% CI for exponential (β).

### Pathway from pulse pressure to dementia

**Figure 3:**
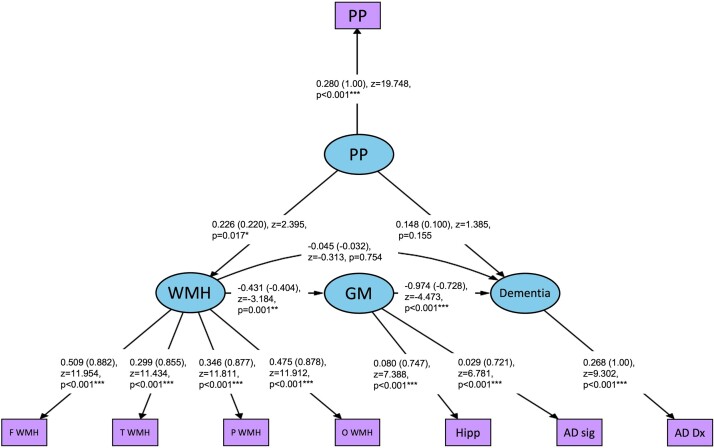
**SEM demonstrating pathway from PP to dementia and intermediate markers.** Standardized beta coefficients are included in parentheses. Paths indicated as significant (*) for *P*-values ≤ 0.05, (**) for *P* ≤ 0.01 and (***) for *P* ≤ 0.001. PP, pulse pressure; WMH, white matter hyperintensities; GM, grey matter; F WMH, frontal WMH; T WMH, temporal WMH; P WMH, parietal WMH; O WMH, occipital WMH; Hipp, hippocampal volume (TIV adjusted); AD sig, Alzheimer’s disease cortical signature thickness, AD Dx, Alzheimer’s disease dementia diagnosis.

There was acceptable overall model fit: *X*^2^ (17, *N* = 195) = 43.622, *P* < 0.001, CFI = 0.947, TLI = 0.912, RMSEA = 0.090 and SRMR = 0.058. However, model fit was weaker when age was included as a covariate, though some indices were still considered acceptable (see Supplemental Fig. 1). Higher PP was related to greater WMH burden (see Fig. 3). In turn, greater WMH volume was associated with increased neurodegeneration. Increased neurodegeneration was subsequently related to dementia. Pulse pressure was not directly associated with dementia. Findings remained when age was added as a covariate to the SEM (see Supplemental Fig. 1).

## Discussion

Higher PP was associated with greater global, parietal and occipital WMH in older adults with DS but not with other markers of cerebrovascular disease. Pulse pressure was not directly associated with dementia status. However, higher PP was indirectly associated with diagnosis of dementia, through WMH and subsequent neurodegeneration.

Blood pressure has not been studied comprehensively in relation to brain or any clinical marker associated with Alzheimer’s disease in adults with DS. To our knowledge, only one previous study examined blood pressure in DS and found that blood pressure did not increase with age in people with DS despite observed age-associated increases in neurotypical participants.^22^ The relationship of PP with cerebrovascular disease was specific to WMH. We did not find any association of PP with PVS, microbleeds or infarcts, suggesting that WMH, a marker of chronic and subtle small vessel disease,^47^ is more sensitive to the impact of relatively higher PP. The other cerebrovascular imaging markers may indicate other mechanistic processes, including impaired glymphatic clearance resulting in PVS,^48^ cerebral amyloid angiopathy that is more frequent in the brains of people with DS and associated with microbleeds^12,49,50^ and large artery disease leading to infarcts.^51^

The lack of direct association of PP with dementia may be explained by the mechanistic chain of events occurring prior to the onset of dementia. For example, higher PP may directly induce cerebrovascular changes rather than act on neurodegenerative processes directly. This possibility is highlighted in the SEM, in which regional WMH burden and neurodegeneration mediated the association between higher PP and dementia diagnosis. The association between WMH and lower cortical thickness and hippocampal volume is supported by our previous work.^39,40^

This study has some limitations. Blood pressure was assessed with a single measurement, which can lower the likelihood of finding robust associations due to measurement reliability. There may have been additional sources of error in the blood pressure measurement that we did not control, including patient- and procedure-related factors (e.g. movement or time of day during measurement). However, these limitations would likely bias our findings towards the null. Despite these concerns, we observed reliable associations of PP with hypothesized outcomes, suggesting that our findings are reliable but may even underestimate the overall effect. Additionally, due to the cross-sectional design, we could not investigate the temporality or causality of the observed relationships. Based on a number of studies highlighting the role of blood pressure as a risk factor for age- and Alzheimer’s disease-related changes in the neurotypical population (see reviews^29,52,53^), it is more likely that higher blood pressure and PP emerge gradually in adulthood and precede associated structural brain changes and subsequent clinical status. The adults with DS included in the study were predominantly non-Hispanic White. In the neurotypical aging population, higher blood pressure is more prevalent among racially and ethnically minoritized populations,^54^ potentially contributing to health disparities among groups. Another future direction is to examine markers other than PP that can more accurately capture arterial stiffness in adults with DS, such as with intracranial pulse wave velocity.^55^

To summarize, we demonstrated that higher PP is associated with both global and posterior WMH burden. In examining a pathway from PP to dementia, we found that PP was related to WMH, which was associated with neurodegeneration, and subsequently to dementia. This work highlights the potential impact of elevated PP in individuals with DS. Monitoring and targeting blood pressure through non-pharmacological or pharmacological approaches may be helpful for people with DS.

## Supplementary Material

fcae157_Supplementary_Data

## Data Availability

Data in the current study came from the Alzheimer’s Biomarker Consortium—Down Syndrome (ABC-DS) study. Data from the ABC-DS can be requested here: https://www.nia.nih.gov/research/abc-ds#data.
