# Supplementary material for: A pathway linking pulse pressure to dementia in adults with Down syndrome
Source: Brain Commun. 2024 May 9;6(3):fcae157. doi: 10.1093/braincomms/fcae157 (PMC11099660; doi:10.1093/braincomms/fcae157)
Supplement: fcae157_Supplementary_Data [file fcae157_supplementary_data.docx]

**SUPPLEMENT**

**Supplemental Table 1.** Sensitivity analyses examining pulse pressure and cerebrovascular imaging markers, excluding participants treated with anti-hypertensive or anti-hypotensive medication.

| **BP Measure** | **Outcome Measure** | ***B*** | **95% CI** | **β** | **t / Wald**^‡^ | **p-value** |
| --- | --- | --- | --- | --- | --- | --- |
| Pulse pressure | Global WMH | 0.061 | (0.004, 0.119) | 0.167 | 2.106 | 0.037 |
|  | Frontal WMH | 0.012 | (-0.015, 0.039) | 0.076 | 0.878 | 0.382 |
|  | Temporal WMH | 0.009 | (-0.001, 0.019) | 0.157 | 1.845 | 0.068 |
|  | Parietal WMH | 0.014 | (0.002, 0.026) | 0.193 | 2.314 | 0.023 |
|  | Occipital WMH | 0.021 | (0.005, 0.038) | 0.200 | 2.583 | 0.011 |
|  | Microbleeds^λ^ | -0.010 | (0.941, 1.042) | 0.990 | 0.150 | 0.698 |
|  | Enlarged perivascular spaces | 0.072 | (-0.012, 0.155) | 0.166 | 1.708 | 0.091 |
|  | Infarcts^λ^ | 0.019 | (0.977, 1.063) | 1.019 | 0.769 | 0.381 |

Sensitivity analysis: excluding participants treated with anti-hypertensive or anti-hypotensive medication.

Separate regression models: Outcome measure ~ pulse pressure + age + sex/gender + scanner type + intercept.

^‡^ A t-value is shown for linear regressions, and a Wald statistic is shown for logistic regressions.

^λ^ Logistic regression was applied for dichotomous outcomes. Β = Exponential (β) and 95%CI = 95% CI for Exponential (β).

**Supplemental Table 2.** Associations of systolic BP, diastolic BP, and MAP with cerebrovascular imaging markers.

| **BP Variable** | **Outcome Marker** | ***B*** | **95% CI** | **β** | **t / Wald**^‡^ | **p-value** |
| --- | --- | --- | --- | --- | --- | --- |
| Systolic BP | Global WMH | 0.036 | (-0.013, 0.084) | 0.113 | 1.456 | 0.148 |
|  | Frontal WMH | 0.004 | (-0.019, 0.027) | 0.027 | 0.318 | 0.751 |
|  | Temporal WMH | 0.007 | (-0.001, 0.015) | 0.143 | 1.743 | 0.084 |
|  | Parietal WMH | 0.008 | (-0.002, 0.018) | 0.132 | 1.630 | 0.106 |
|  | Occipital WMH | 0.013 | (-0.001, 0.026) | 0.138 | 1.825 | 0.071 |
|  | Microbleeds^λ^ | -0.022 | (0.936, 1.023) | 0.978 | 0.938 | 0.333 |
|  | Enlarged Perivascular spaces | 0.049 | (-0.023, 0.121) | 0.126 | 1.359 | 0.177 |
|  | Infarcts^λ^ | 0.012 | (0.975, 1.050) | 1.012 | 0.529 | 0.529 |
| Diastolic BP | Global WMH | -0.014 | (-0.090, 0.062) | -0.029 | -0.361 | 0.719 |
|  | Frontal WMH | -0.012 | (-0.048, 0.024) | -0.056 | -0.658 | 0.512 |
|  | Temporal WMH | 0.003 | (-0.010, 0.016) | 0.039 | 0.463 | 0.644 |
|  | Parietal WMH | -0.002 | (-0.017, 0.013) | -0.022 | -0.268 | 0.789 |
|  | Occipital WMH | -0.002 | (-0.024, 0.020) | -0.013 | -0.174 | 0.863 |
|  | Microbleeds^λ^ | -0.030 | (0.905, 1.040) | 0.970 | 0.732 | 0.229 |
|  | Enlarged perivascular spaces | -0.015 | (-0.123, 0.092) | -0.027 | -0.283 | 0.777 |
|  | Infarcts^λ^ | -0.009 | (0.938, 1.047) | 0.991 | 0.109 | 0.741 |
| Mean Arterial Pressure | Global WMH | 0.018 | (-0.055, 0.091) | 0.039 | 0.490 | 0.625 |
|  | Frontal WMH | -0.005 | (-0.039, 0.030) | -0.022 | -0.261 | 0.795 |
|  | Temporal WMH | 0.007 | (-0.005, 0.019) | 0.097 | 1.161 | 0.248 |
|  | Parietal WMH | 0.005 | (-0.010, 0.019) | 0.052 | 0.635 | 0.527 |
|  | Occipital WMH | 0.008 | (-0.012, 0.029) | 0.061 | 0.789 | 0.431 |
|  | Microbleeds^λ^ | -0.037 | (0.901, 1.032) | 0.964 | 1.119 | 0.290 |
|  | Enlarged perivascular spaces | 0.025 | (-0.081, 0.131) | 0.044 | 0.471 | 0.638 |
|  | Infarcts^λ^ | 0.003 | (0.949, 1.059) | 1.0039 | 0.009 | 0.925 |

Separate regression model: Outcome measure ~ BP measure + age + sex/gender + scanner type + intercept.

^‡^ A t-value is shown for linear regressions, and a Wald statistic is shown for logistic regressions.

^λ^ Logistic regression was applied for dichotomous outcomes. β = Exponential (β) and 95%CI = 95% CI for Exponential (β).

**Supplemental Figure 1.** Structural equation model demonstrating pathway from pulse pressure to dementia and intermediate markers when age is included as a covariate.

**
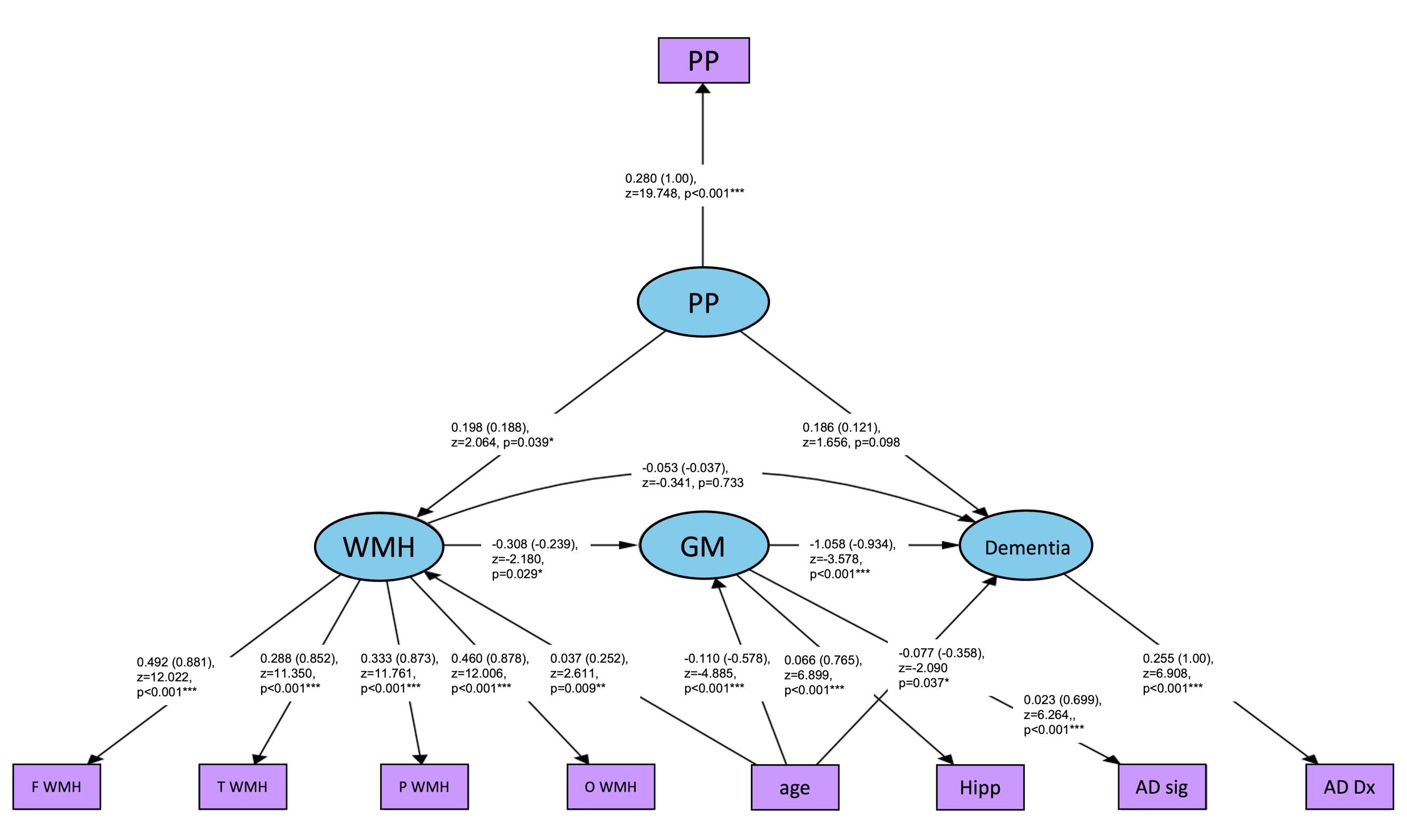
**

Standardized beta coefficients are included in parentheses.

Paths indicated as significant (*) for p-values < 0.05, (**) for p < 0.01, and (***) for p < 0.001.

PP: pulse pressure, WMH: white matter hyperintensities, GM: gray matter, F WMH: frontal WMH, T WMH: temporal WMH, P WMH: parietal WMH, O WMH: occipital WMH, Hipp: hippocampal volume (TIV adjusted), AD sig: Alzheimer’s disease cortical signature thickness, AD Dx: Alzheimer’s disease dementia diagnosis. Model fit indices: *X^2^* (22, *N*=195) = 63.834, *p* < 0.001, CFI = 0.925, TLI = 0.877, RMSEA = 0.099, and SRMR = 0.059.
